# AptaGUI—A Graphical User Interface for the Efficient Analysis of HT-SELEX Data

**DOI:** 10.1038/mtna.2015.26

**Published:** 2015-10-13

**Authors:** Jan Hoinka, Phuong Dao, Teresa M Przytycka

**Affiliations:** 1 National Center for Biotechnology Information, National Institutes of Health, Bethesda, Maryland, USA

**To the Editor:** Revolutionizing the traditional Systematic Evolution of Ligands by EXponential Enrichment (SELEX), high-throughput SELEX (HT-SELEX) has become the method of choice for the identification of aptamers—synthetic, single-stranded (ribo)nucleic molecules that bind to a given molecular target.^1^ In contrast to traditional SELEX, in which only data from the last selection cycle is obtained, HT-SELEX allows for sequencing of every round (including control cycles) which are then jointly analyzed and interpreted.

However, given the vast amount of data generated by this technology, purely text-based tools to visualize and manipulate aptamers have proven impractical and time consuming for many researchers in this field. Currently, COMPAS^2^ by AptaIT is the only pipeline known to us to provide graphical navigation of HT-SELEX data but is licensed as proprietary software and only obtainable in conjunction with the purchase of an *in vitro* selection from the same company.

We report AptaGUI, a new open-source and platform-independent graphical user interface (GUI) for the dynamic visualization of HT-SELEX data. In particular, AptaGUI includes support for applications of our AptaTools package (http://www.ncbi.nlm.nih.gov/CBBresearch/Przytycka/index.cgi#aptatools) that contains many useful algorithms for HT-SELEX analysis, including but not limited to data pre-processing, tracking the changes of individual aptamers, as well as entire aptamer family (groups of aptamers sharing highly similar nucleotide sequences), throughout selection cycles. This includes computing cycle-to-cycle enrichment of aptamers and their families and displaying them according to the user's specification. AptaGUI provides researchers with intuitive multi-scale access to any desired aspect of a SELEX experiment, ranging from general statistics such as quality control measures, nucleotide distributions, and diversity analysis of the individual rounds, through the intermediate representation of aptamer families as sequence logos, to detailed information on individual aptamers and their properties. It thus allows for capturing potential issues with the selection protocol, identification of candidate aptamers, and their detailed sequence and structure-based analysis among others.

AptaGUI currently features four main sections focusing on (i) data quality control, (ii) experimental details such as enrichment statistics throughout selection cycles, (iii) sequence-based analysis, and (iv) aptamer family (cluster)–based analysis. In addition, AptaGUI provides secure, global, multi-user access to the data sets, hence enabling real-time, collaborative studies in the field of aptamer discovery.

AptaGUI is written in Java version 8 and uses a MySQL database containing all results produced by our software package AptaTools. AptaTools includes, in addition to basic analysis tools, the clustering algorithm AptaCLUSTER and the mutant analysis utility AptaMut.^3,4^ The interface is tabulated into four sections denoted as Overview, Experiment Details, Sequence Relations, and Cluster Relations, each of which corresponding to a fundamental aspect of aptamer-related data analysis as summarized below.

The Overview tab displays general information about a selected experiment, including but not limited to quality control reports and selection round details (**Figure 1**, top panel). In terms of quality control, it provides several statistics regarding the consistency of paired-end sequence reads, demultiplexing the reads into the different selection rounds and extracting the randomized region. In terms of selection round details, for each sequenced cycle, tag–primer combinations used during selection and sequencing, the pool size, the pools nucleotide distribution, and detailed information regarding the cycles nucleotide composition for the forward reads, reverse reads (if paired-end data was used), and the randomized regions that passed the demultiplexing and quality control filters is displayed. Finally, for each round, the option to view all aptamers as well as aptamer families and control cycles (should these be available) is also available. Taken together, the data summarized in this tab serves as a first step to quickly assess the overall quality of not only the raw sequencing data, but also the quality of the selection process itself.

The Experiment Details tab provides information regarding global selection properties of the SELEX experiment for both sequences and aptamer families. For sequences, it summarizes the distribution of singletons (aptamers with a count of 1), the frequency of enriched species, as well as the percentage of distinct aptamers that make up each pool. Equivalently, for aptamer families (clusters), the average cluster size and diversity (*i.e.*, the number of unique aptamers per family), as well as the number of clusters per pool are displayed. These pool properties allow for progress assessment of the SELEX experiment and provide insight into the exponential enrichment aspects of target-affine species in the pool.

The Sequence Relations tab retrieves detailed information regarding sequence count (the frequency of the aptamer), fraction (the percent of this aptamer with respect to the pool size), and enrichment (fold change in fraction between consecutive cycles) for every aptamer in the experiment and for every selection cycle present in the experiment (**Figure 1**, center panel). The enrichment values between consecutive selection cycles can also be plotted graphically as a function of the cycle number allowing for capturing cycle-specific enrichment dynamics. In addition, aptamer sequences can be queried for occurrences of a particular motif (including wild-card searches). The results are displayed according to the user's specifications including sorting aptamers of a specific cycle by count, fraction, or enrichment, allowing for easy accesses to aptamers of interest. AptaGUI also supports the prediction and graphical presentation of secondary structures for single aptamers as well as aptamer families (consensus structures) via the integration of PPFold^5^ and VaRNA^6^ into the software. Furthermore, each sequence can be annotated with custom information in real time for collaborative editing and analysis of a dataset across multiple platforms. Finally, candidate sequences can be exported (with or without primers) should any kind of post-processing by third party software be required.

The Cluster Relations tab provides detailed information about aptamer families identified by AptaCLUSTER and their relation throughout the selection cycles (**Figure 1**, bottom panel). For each family, the cluster identified in the last selection round and the corresponding clusters from all previous selection cycles are shown. Each cluster is summarized by its sequence logo and, in analogy to the properties shown in the Sequence Relations tab, displays its size (total number of sequences), pool fraction (the percentage the cluster occupies in the corresponding selection round), the cluster diversity (number of unique sequences in the cluster), as well as the clusters enrichment. The cluster enrichment values can be analyzed graphically in order to identify aptamer families of potential interest. Additional information for each cluster, including a list of its comprising sequences, the distribution of their counts, and a comprehensive analysis of the single-nucleotide variations present in the cluster is available by clicking the Show Sequences button. Additionally, the user has the option to sort the clusters based on size, unique sequences, or enrichment and to search for particular clusters containing a specific, possibly gaped, sequence motif. Finally, a global overview of the distribution of cluster sizes (cluster size versus their frequency of occurrence) for all selection rounds is also provided.

A large number of additional features, such as the analysis of mutants within each cluster via AptaMut,^2^ are described in the user manual available online.

AptaGUI provides the first open source graphical, multi-user, and platform-independent navigation for high-throughput sequencing data from HT-SELEX experiments and facilitates the identification and analysis of aptamers through its interactive capabilities.

## Figures and Tables

**Figure 1:**
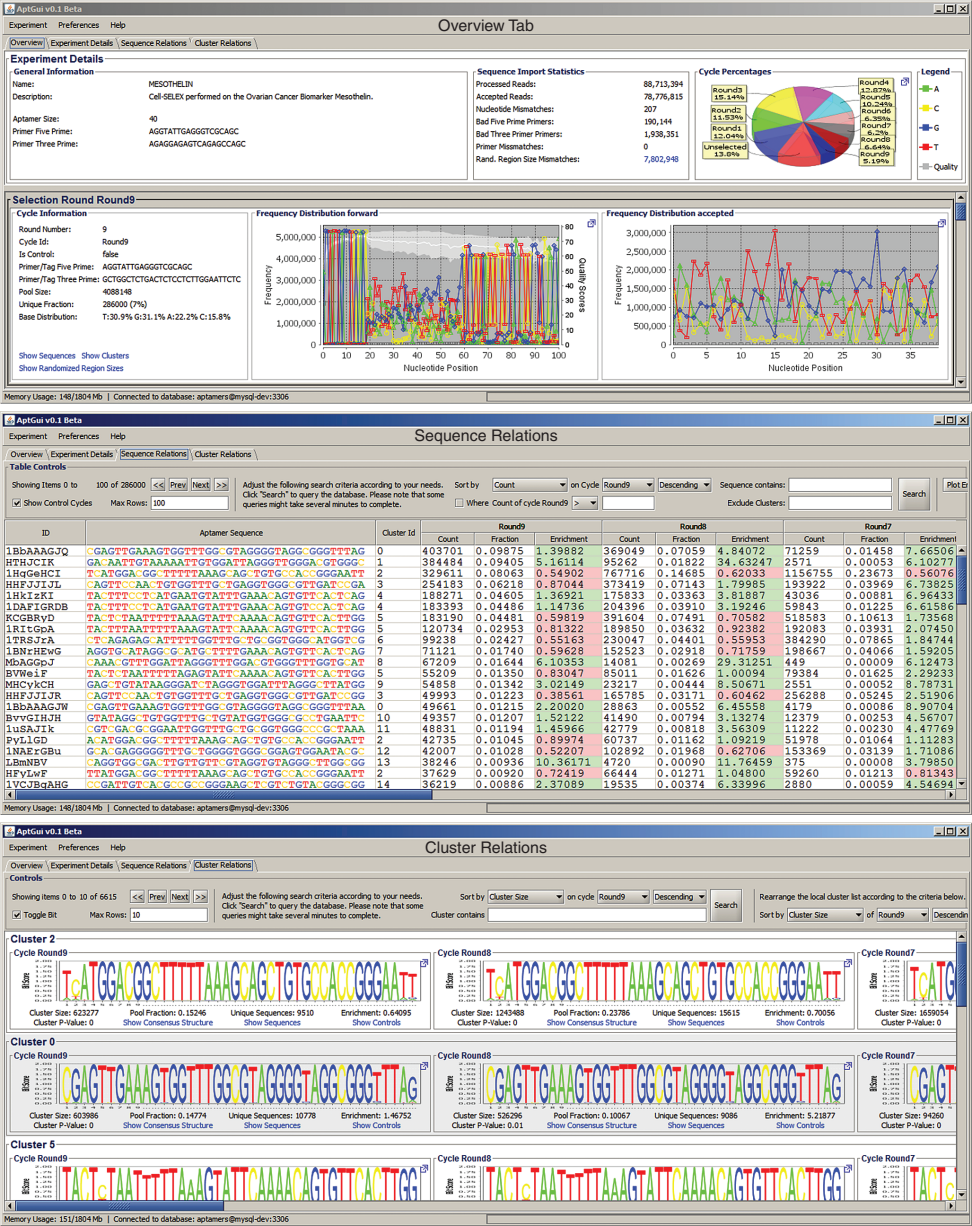
Screenshots of AptaGUI showing the Overview tab (top), the Sequence relations tab (center), and the Cluster relations tab (bottom).
